# Unusual Vascular Distribution in the Third Segment of the Axillary Artery

**DOI:** 10.3390/medicina59050913

**Published:** 2023-05-10

**Authors:** Daniel Ramos-Alicea, Jordan Marcano-Anaya, Mario Loomis, Norman Ramirez, Jailenne I. Quiñones-Rodríguez

**Affiliations:** 1 Department of Anatomy and Cell Biology, School of Medicine, Universidad Central del Caribe, Bayamon, PR 00960, USA; 2 Department of Clinical Anatomy, College of Osteopathic Medicine, Sam Houston State University, Conroe, TX 77304, USA; 3 Department of Pediatric Orthopedic Surgery, Mayaguez Medical Center, Mayaguez, PR 00960, USA

**Keywords:** third segment axillary artery, posterior circumflex humeral artery, subscapular artery, latissimus dorsi flap procedures, rotator cuff surgery, proximal humeral fracture

## Abstract

The third segment of the axillary artery (TSAA) is the main vascular supply to the muscles of the upper limb. Numerous studies have reported atypical branching patterns of the TSAA, which can complicate operative interventions involving structures supplied by this segment of the artery. Our current study evaluated a previously undescribed branching pattern in the TSAA, in which the subscapular artery gave rise to an unusual posterior humeral circumflex artery, and a second subscapular artery. In addition, a third variant was found in the origin of the thoracodorsal artery: two collateral horizontal arteries supplying the deep medial surface of the latissimus dorsi muscle. Vascular anatomical variants may affect the classical upper limb interventions requiring modification of the traditional surgical approaches. This case report aims to evaluate these variants from a clinical perspective regarding the management of upper limb trauma, axillary, breast, and muscle flap surgery.

## 1. Introduction

**Figure 1:**
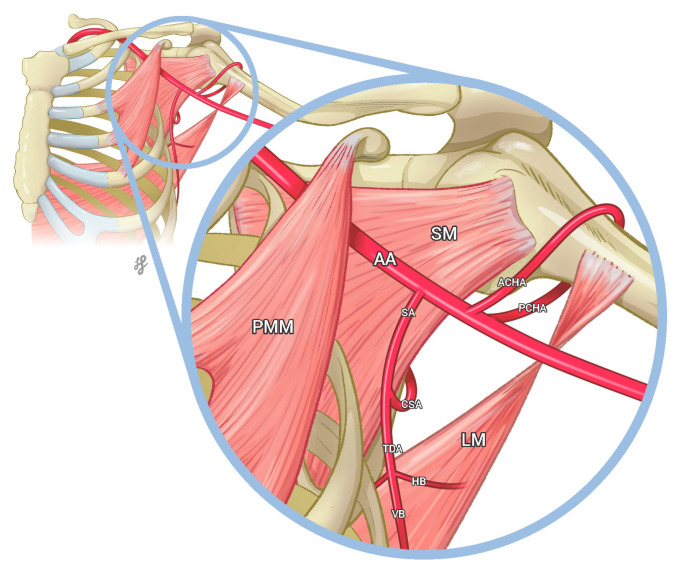
Illustration showing the classically described third segment of the axillary artery distal to the pectoralis minor muscle (PMM), giving off the subscapular artery (SA), which travels caudally, shortly after which it bifurcates to give the circumflex artery (CSA), and the thoracodorsal artery (TDA). The CSA curves posteriorly around the lateral border of the scapula, passing posteriorly between the subscapularis muscle (SM) and the teres major. Then, the TDA continues the course of the SA, descending and dividing into a vertical branch (VB) and a horizontal branch (HB), entering the apex of the latissimus dorsi muscle (LM). Finally, the circumflex humeral arteries, the posterior circumflex humeral artery (PCHA), and the anterior circumflex humeral artery (ACHA) encircle the surgical neck of the humerus, anastomosing with each other. Printed with permission from Lucia Garces © 2023.

The subclavian artery continues through the upper arm to become the axillary artery as it passes through the lateral border of the first rib. At the same time, the axillary artery is bordered inferiorly by the teres major muscle. On its course, this artery typically gives off six branches that supply the lateral thorax, the axillary region, and the upper limb. The six branches are divided into three segments (first, second, and third), established by the pectoralis minor muscle (Figure 1) [1].

The main arterial vessels originating from the heart begin forming around the embryo’s third week of gestation. By this time, the vessels include the aortic arches. Of all the aortic arches, the fourth aortic arch gives rise to the seventh intersegmental artery, which later forms the subclavian artery, and continues as the axillary artery in the upper limb [2]. Anomalies in branching arterial patterns arise from these embryological divisions during development. It has been suggested that the Tbx1 gene promotes an incongruous formation in the course of the fourth arch’s arteries within an interruption in the aortic arch [3].

Usually, in humans, the second and third segments of the axillary artery exhibit the most common vascular variation, where five of six arteries displayed a higher frequency of anatomical disturbances [4]. One of the most common variations reported is a common trunk for the lateral thoracic artery and the subscapular artery, accompanied with a high prevalence rate (37.2%) [4]. This variation is implicated in a higher risk of critical limb ischemia in the case of a thrombus or embolus lodging distally to the second segment of the axillary artery [4], thereby emphasizing the importance of recognizing these arterial variations. Other frequently described variations in the literature are those of the anterior and posterior humeral circumflex arteries. The posterior circumflex humeral artery (PCHA) is atypically found as a single branch, with the anterior circumflex humeral artery (ACHA) stemming from it [5]. The atypical branching patterns of these arteries are implicated with an increased incidence of necrosis in proximal humeral fractures [5]. However, a variation in the PCHA which enables it to traverse the quadrangular space, makes it less susceptible to sports injuries [5,6]. Details regarding the deviation from the typical arterial pattern are crucial for anatomists, plastic and orthopedic surgeons, vascular radiologists, and interventional cardiologists, among others [7,8,9,10,11].

The present study aims to describe an atypical branching of the TSAA regarding the course of the PCHA, a second branch from the SSA, and an unexpected proximal collateral originating from the thoracodorsal artery (TDA). Finally, we will establish the clinical significance of such variations to different surgical specialties.

## 2. Case Report

During a routine donor dissection, several anatomical variations were observed in an elderly adult female cadaver. The unilateral variations were seen on the left-branching patterns of the TSAA. The clinical history, family history, and cause of death were unremarkable.

**Figure 2:**
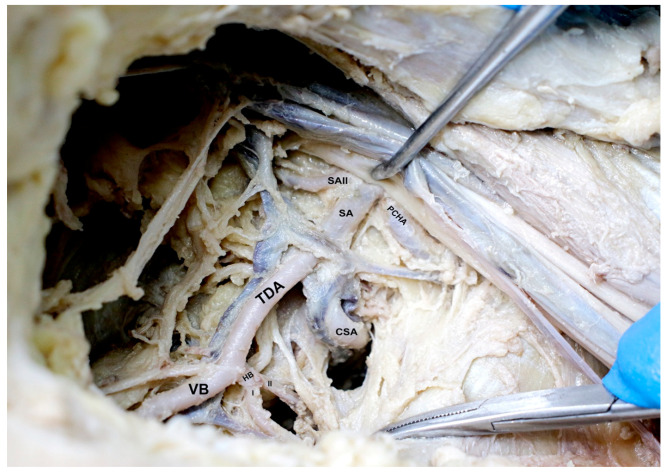
Vascular atypical origins of the third part of the axillary artery. The subscapular artery (SA) is divided into subscapular artery II (SAII) and the posterior circumflex humeral artery (PCHA). Following the course, the circumflex subscapular artery (CSA) is on the distal margin, and medially, the terminal branch, and the thoracodorsal artery (TDA). The TDS gives rise to two horizontal branches (HB: I, II), and a vertical branch (VB).

**Figure 3:**
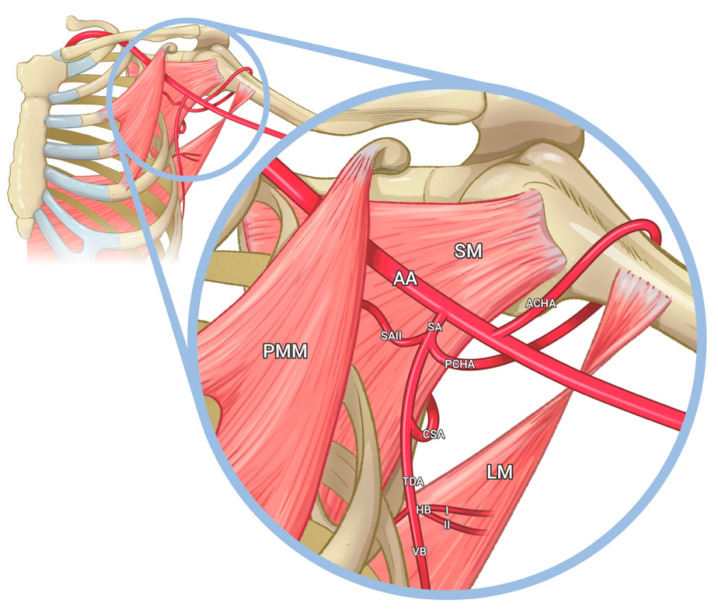
Illustration depicting the abnormal variation found on the third part of the axillary artery. The SA originated from the axillary artery, distally to the pectoralis minor muscle (PMM). It is divided into the SAII following its course to the posterior side to supply the subscapularis muscle (SM) and the PCHA. The PCHA and the anterior circumflex humeral artery (ACHA) form anastomosis to supply the humeral head. Then from the SA, the CSA is located distally, and medially, the terminal branch of the SA, and the TDA. The TDS gives rise to two interconnections horizontally and vertically, predominantly supplying the latissimus dorsi (LM): HBI, HBII, and the VB. Printed with permission from Lucia Garces © 2023.

An atypical origin of the PCHA was observed from the subscapular artery lateral to its origin (Figure 2). After its origin, the PCHA was accompanied by the axillary nerve, descending beneath the subscapularis muscle towards the quadrangular space to supply the posterior shoulder joint and muscles. Another variation regarding the opposite medial side of the subscapular artery was identified. The subscapular artery revealed an unusual branch with an apparent course to supply the anterosuperior portion of the subscapularis muscle. This branch, which we categorized as the subscapular artery II (SAII), originates from the subscapular trunk at the same level as the PCHA vascular variant. Then, the SAII courses deeper medially into the axillary region, bifurcating to supply the subscapularis muscle. Following down to the thoracodorsal artery, an uncommon pattern was observed regarding the supply of the latissimus dorsi muscle. Two horizontal branches (HBI and HBII) were identified originating from the thoracodorsal artery, supplying the superomedial fibers of the latissimus dorsi muscle (Figure 3).

## 3. Discussion

### 3.1. Gross Anatomy

Atypical branches are titled after their origin, their relationship with close anatomical structures, and their anatomical locations. We reported a series of variations named according to their origin and blood supply. Studies published in the medical literature have documented many axillary artery variations, and more frequently of those arteries branching from the third segment [6]. These findings included a common trunk with the PCHA [7]. Another study reported that the PCHA branched off the SSA in up to 30% of cases from the TSAA [12].

This study reports a previously undescribed first branch of the TSAA giving off the SSA. Classically, the third segment branches into the SSA and the posterior and anterior circumflex arteries separately. However, examination of the left arm presented an unusual branching of the SSA, which gave off the PCHA and a second smaller SSA (SAII) branch. On the other hand, the TDA usually supplies the latissimus dorsi muscle with its horizontal and vertical branches. However, examination of the TDA in the left arm exhibited a horizontal extension (HBI and HBII), providing vascular supply to the latissimus dorsi muscle. Pathologies affecting the SAII might become an obstacle in surgical repair. Failure to identify variant anatomy is a commonly cited technical error in surgical injuries, even among experienced surgeons [4].

### 3.2. Clinical Significance

#### 3.2.1. Plastic Surgery—Viewpoint

These anatomical variations have clinical implications for breast surgery, axillary surgery, and pedicled and free muscle flap surgery. Plastic surgery, named after the Greek term, “Plastikos”, meaning to shape or mold, often moves tissue from a healthy area to reshape abnormalities, such as in congenital breast asymmetries, or to cover deformities from trauma, cancer surgeries, or other abnormalities. In all of these circumstances, the blood supply to the tissue being moved is paramount. During an axillary node dissection following a mastectomy for breast cancer, or a resection of melanoma from a region draining into the axillary basin, there is a “safe” zone below the axillary artery and between the thoracodorsal and lateral thoracic arteries, from which the node-bearing tissue is removed. Significantly, the SAII variation described herein crosses directly through this zone, and as such would be in danger of being injured during an axillary dissection. Success of the latissimus dorsi flap, utilized in breast reconstruction both as a pedicled and as a free flap, is dependent upon the knowledge of the vascular anatomy [13]. Anatomical variations have significant implications for latissimus dorsi flap procedures (LDFP) [14]. In LDFP, surgeons undergo defect analysis, observing the areas requiring reconstruction for vascular status, infections, previous surgeries, malignancies, and other circumstances. As vascular perfusion of the transferred tissue is crucial for the flap design as well as its survival, the development of the flap techniques has depended on defining the vascular anatomy of the skin and the underlying tissue [15].

In addition to the SAII artery variation affecting axillary node dissection, we report an additional horizontal branch from the thoracodorsal artery that can influence the planning of a pedicled or free latissimus dorsi muscle flap. Usually, the vascular pedicle is mobilized from the muscle, dividing small branches, if necessary. The significant-sized branch reported here, near the origin of the latissimus dorsi muscle would therefore be problematic. When a latissimus dorsi muscle is used as a pedicled flap, the muscle’s insertion is often released, and the flap is maintained only on its long vascular pedicle. These atypical horizontal branches, which arise proximally, would tether the muscle to the proximal portion of the vessel, thereby limiting its reach. Likewise, when the muscle is used as a free flap, a long vascular pedicle is necessary to be sure that the site of anastomosis is well outside the zone of injury. Such zones of injury would include complex fracture sites and areas of previous radiation therapy. A short pedicle would force the anastomosis to be within the zone of injury and therefore be significantly more prone to thrombosis and flap failure. Similar to other systems, damage to the arterial system of the latissimus dorsi has a spectrum of presentations. For example, those at a proximal level present with severe bleeding and require emergency surgical intervention to control the resulting hemorrhage and revascularize the muscle [16,17,18]. With all flap transfers, if the vascular pedicle is not functional, there will be a greater risk of muscle necrosis.

Since the latissimus dorsi muscle is generally very well-perfused, the LDFP are considered very robust, and preoperative vascular studies are not usually required. Should this variation be encountered intraoperatively, however, the plan would have to be modified, using only the lower portion of the muscle to maintain a long pedicle, and this may still be insufficient. To therefore optimize surgical planning, it would be wise to rule out this segmental vascular supply that we describe and ensure that the entire muscle will be available for reconstruction with a sufficiently long pedicle. For this, selective arteriography or CT angiography is recommended to determine the patency of the planned flap pedicle [19]. Also, previous examination and history for obesity, tobacco use, hypertension, immunosuppression, and pulmonary compromise must be evaluated [17]. These will influence the patient’s condition and the flap selection, as well as the success of the flap surgery. Disregarding such systemic factors may increase the risk of thromboembolism [20,21,22].

#### 3.2.2. Orthopedic Surgery—Viewpoint

The subscapular trunk is typically the axillary artery’s largest branch. This trunk provides an extension that serves as the most critical blood supply of the subscapularis muscle, the circumflex scapular artery [2]. Our case presents an unusual dual blood supply to the subscapularis muscle.

The subscapularis muscle is supplied classically by the subscapular, suprascapular, and lateral thoracic arteries. Knowledge regarding the blood supply to this muscle is essential because of its large size relative to the rest of the rotator cuff muscles [23]. Awareness of the anatomical variations in this region is essential for rotator cuff injury treatments to prevent unexpected errors in orthopedic surgery, for example, transections of the thoracoacromial artery in an acromioplasty [24]. The subscapularis tendon makes up the anterior portion of the cuff and balances the shoulder, thereby decreasing the strain on rotator cuff repairs. Pathologies involving variant blood supply are rare, however, recognizing such atypical vascular presentations may prevent complications in the treatment of rotator cuff injuries, axillary aneurysms, and thrombosis [25].

Opposite to the SAII, we found an abnormal origin of PCHA. Usually, the PCHA arises from the TSAA posterior to the origin of the ACHA [26]. This artery leaves the axilla and courses through the quadrangular space along the axillary nerve and its associated vein. Eventually, the PCHA divides into its terminal branches and wraps around the humeral neck. At this point, it provides blood supply to the humeral head, shoulder joint, and muscles. Studies suggest that variations in the origin of this artery have clinical relevance [19,23,26]. Medical literature emphasizes the vascular variant of quadrilateral space syndrome (QSS), aneurysms, and proximal humeral fracture-dislocation. In QSS, the PCHA is compressed when passing through the quadrangular space. This stress leads to the humeral head and muscle ischemia. This condition is particularly prevalent in athletes who exert overhead arm stress [26]. Repeated damage to this vessel can cause wall weakening, leading to thrombosis, subsequent embolism, and even aneurysms [27,28]. This syndrome often goes undiagnosed, so it is therefore crucial to recognize the typical and atypical anatomy of the PCHA [29].

On the other hand, proximal humeral fracture-dislocation increases the risk of avascular necrosis of the humeral head. Mainly, this complication can result from damage to the PCHA. Medical literature reports that 64% of the blood supply to the humeral head is provided by this vessel [30]. Furthermore, during rotator cuff surgery, iatrogenic injury can damage the PCHA. This complication is common when using anchors during surgery that may impair the blood supply [24]. Therefore, comprehension of the variant origin and course of the PCHA is of the utmost importance for repairing and preventing vascular damage.

## 4. Conclusions

Knowledge of the vascular variants throughout the body highlights clinical and surgical relevance, as reported above. In addition, the importance of reporting on these anatomical variations can assist in modifying surgical approaches and patient management and prevent possible complications in specific patient populations. In this case, an unreported multi-variant TSAA has significant relevance to diverse medical specialties, including general, orthopedic, and plastic surgery. Describing new anatomical variants is critical to improve the surgical outcomes and prevent iatrogenic injuries by physicians.

## Data Availability

Not applicable.
